# Altered lignification in *mur1-1* a mutant deficient in GDP-L-fucose synthesis with reduced RG-II cross linking

**DOI:** 10.1371/journal.pone.0184820

**Published:** 2017-09-29

**Authors:** Aline Voxeur, Ludivine Soubigou-Taconnat, Frédéric Legée, Kaori Sakai, Sébastien Antelme, Mylène Durand-Tardif, Catherine Lapierre, Richard Sibout

**Affiliations:** 1 Institut Jean-Pierre Bourgin, INRA, AgroParisTech, CNRS, Université Paris-Saclay, Versailles, France; 2 Institute of Plant Sciences Paris Saclay IPS2, CNRS, INRA, Université Paris-Sud, Université Evry, Université Paris-Saclay, Bâtiment, Orsay, France; University of Massachusetts Amherst, UNITED STATES

## Abstract

In the plant cell wall, boron links two pectic domain rhamnogalacturonan II (RG-II) chains together to form a dimer and thus contributes to the reinforcement of cell adhesion. We studied the *mur1-1* mutant of *Arabidopsis thaliana* which has lost the ability to form GDP-fucose in the shoots and show that the extent of RG-II cross-linking is reduced in the lignified stem of this mutant. Surprisingly, *MUR1* mutation induced an enrichment of resistant interunit bonds in lignin and triggered the overexpression of many genes involved in lignified tissue formation and in jasmonic acid signaling. The defect in GDP-fucose synthesis induced a loss of cell adhesion at the interface between stele and cortex, as well as between interfascicular fibers. This led to the formation of regenerative xylem, where tissue detachment occurred, and underlined a loss of resistance to mechanical forces. Similar observations were also made on *bor1-3* mutant stems which are altered in boron xylem loading, leading us to suggest that diminished RG-II dimerization is responsible for regenerative xylem formation.

## Introduction

Plant cell walls are crucial for many aspects of plant development. The plant primary cell wall is mainly composed of cellulose, hemicelluloses, and pectins, that impact both strength and flexibility and thus determine plant shape. Neighboring cells and their cell walls are connected by a pectin-rich middle lamella that is involved in cell adhesion [1](Daher and Braybrook, 2015). During expansion, polysaccharides of primary cell wall are continuously remodeled. In some specific tissues such as water conducting tracheary elements and structurally important fibers, the cessation of growth coincides with the deposition of a high concentrations of lignin, and lignification begins in the primary cell wall and continue during secondary cell wall formation.

In eudicotyledonous plants, the secondary cell wall lignin polymer is predominantly made from coniferyl and sinapyl alcohols, but also from some traces of *p*-coumaryl alcohol. These three monolignols give rise, respectively, to guaiacyl, syringyl, and *p*-hydroxyphenyl units in the lignin polymer. These lignin units are linked via labile arylglycerol–β–aryl ether linkages (referred to as β–*O*–4 bonds) or via more resistant carbon-carbon or biphenyl ether bonds [2],[3]. Lignin in the pectin-rich middle lamella is enriched in guaiacyl and hydroxyphenyl units as well as in resistant inter-unit bonds [4],[5].

Pectins are the most complex class of polysaccharides in plant cell wall [6] and are composed of three main blocks: homogalacturonans, rhamnogalacturonan I (RG-I), and RG-II. The complex RG-II polysaccharide comprises an α-1,4-linked homogalacturonan backbone substituted with four to six structurally different oligosaccharide side-chains [7],[8]. Much of the cell wall RG-II occurs as dimers cross-linked by a borate di-ester via the *cis-*diol groups of two apiose residues of side-chain A [9].

**Fig 1:**
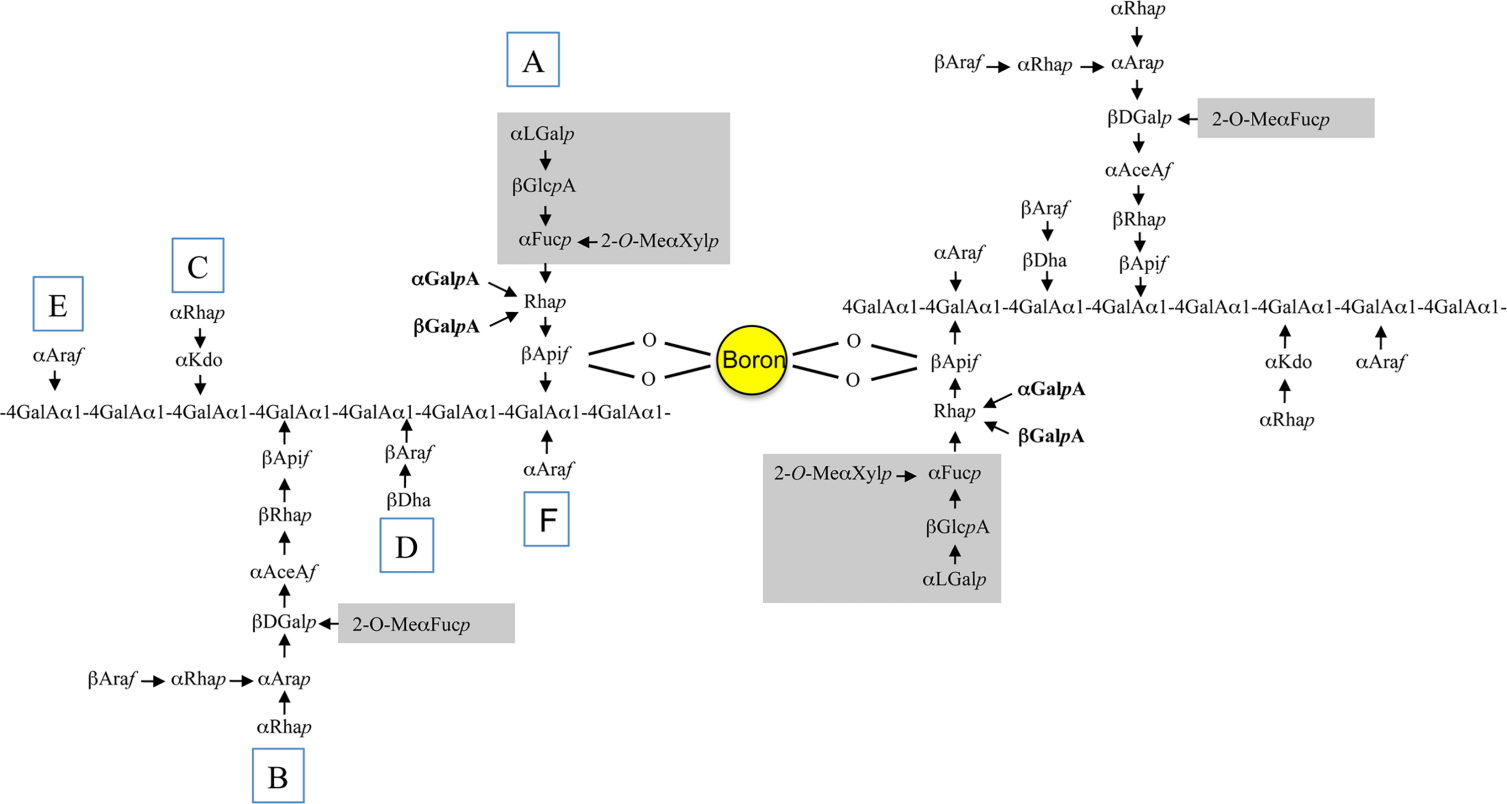
Structure of RG-II and diester linkages with boron. The RG-II dimer is formed by ester bonds between a boron atom and the apiosyl residues of side chain A. The grey part of the side chain A is absent in *mur1-1* RG-II, a defect that leads to a reduced RG-II dimer formation [13],[14]. Rha: rhamnose; Fuc: fucose; Araf: arabinofuranose; Ara: arabinopyranose; Ace: aceric acid; GalA: galacturonic acid; Gluc: glucuronic acid; Api: apiose; Dha: 2-keto-3-deoxy-D-*lyxo*-heptulosaric acid; Kdo: 2-*keto*-3-deoxy-D-manno-octulosonic acid; Gal: galactose; Xyl: xylose.

Interestingly, some authors suggested that the ability of vascular plants to maintain upright growth and to form lignified secondary walls may be correlated with the presence of boron in the cell wall [10],[11],[12]. The RG-II structure is highly conserved between species [11] although relatively minor variations have been reported [13]. Indeed the modification of its monosaccharide composition decreases its ability to dimerize, potentially inducing defects in intercellular attachment in non-lignified tissues that are accompanied by severe growth defects [13],[14],[15],[16],[17],[18]. For instance, it was recently confirmed that the loss of galactose-glucuronic acid (αL-Gal→βD-GlcA) present on side chain A destabilizes the interaction of apiose with borate [8]. Similarly, the *mur1-1* arabidopsis mutant deficient in GDP-fucose synthesis [19] has a 50% decrease in RG-II dimerization due to side-chain A modification [20] (Fig 1). Interestingly enough, the fragile elongating stem of *mur1-1* and its dwarf phenotype can be rescued by addition of boric acid [14],[21]. Likewise, it is noteworthy that mutants for boron transporters have a dwarf phenotype caused by lower RG-II cross linking [22],[23].

The impact of pectin modifications on lignification has been poorly investigated until now. In the present work, we investigated how the loss of function in the synthesis of GDP-fucose impacts RG-II cross linking and lignification in *mur1-1* mature inflorescence. This study revealed misregulation of lignin-related genes together with alterations in lignin structure of the *mur1-1* stem. We also observed defects in inter-cellular attachment that appear to be associated with the induction of traumatic lignified tissues and regenerative xylem cells.

## Material & methods

### Plant materials and growth conditions

All the *Arabidopsis thaliana* L. plants used were from the Columbia background. Seeds from *mur1-1* and *mur1-2* mutants were obtained from the Arabidopsis Biological Resource Center at the Ohio State University (Columbus). Seeds from *bor1-3* mutants were a kind gift of K. Miwa [23]. All cultures were performed in long-day conditions (16/8 h light/dark) in a greenhouse. Plants were grown in soil (St-Mix-Sable from Jiffy-Tref B. V. Netherlands) containing fertilizer (1.20 kg/m^3^ with NPK 06:14:27). Plants were watered weekly with a commercial nutrient solution (FERTIL, Plant Product Co. Canada) diluted at 2.5 g/L and containing NO3 (11%), NH3 (4%), P_2_O_5_ (10%), K_2_O (30%), boric acid (0.02%), EDTA-chelated copper (0.05%), EDTA-chelated Fe (0.10%), EDTA-chelated Mn (0.05%), EDTA-chelated Mo (0.0012%), EDTA-chelated Zn (0.05%). For rescue experiments of *mur1-1*, plants were spayed twice a week (during three weeks) with a boron acid solution (5 mg/mL) as soon as the floral stem emerged. For chemical studies, stems from mature dried plants (80–100 days old) were used.

### Monosaccharide composition

Neutral monosaccharide composition was determined on 5 mg of dried alcohol-insoluble material after hydrolysis in 2.5 M trifluoroacetic acid for 1.5 h at 100°C as described previously [24]. To determine the cellulose content, the residual pellet obtained after the monosaccharide analysis was rinsed twice with 10 volumes of water and then hydrolyzed with H_2_SO_4_ as described by Updegraff [25]. The released monosaccharides were diluted 500-fold and then quantified using high-performance anion-exchange chromatography-pulsed-amperometric detection as described previously [24].

### Lignin content and structure determination

The Klason lignin content was measured according to Dence [26]. Lignin structure was studied by thioacidolysis, as described previously [27]. The lignin-derived thioacidolysis monomers were identified by gas chromatography-mass spectrometry as their trimethylsilylated derivatives. All the analyses were performed with at least three biological replicates. Significant differences were inferred by a Kruskal-Wallis one way test (*P* < 0.05).

### Analytical pyrolysis

The extractive-free cell wall samples were subjected to pyrolysis-gas chromatography-mass spectrometry analyses using a CDS model 5250 pyroprobe autosampler (CDS Analytical, Inc., Oxford, PA, USA) interfaced to an Agilent 6890/5973 gas chromatography-mass spectrometry (GC-MS, Agilent Technologies Inc, Bellevue, WA, USA). The samples were pyrolyzed in a quartz tube and at 500°C for 10 s, using helium as the carrier gas with a flow rate of 1 mL/min. The volatile pyrolysis products were separated on a GC capillary column (5% phenyl methyl siloxane, 30 m, 250 μm i.d., 0.25 μm film thickness, Model Agilent 19091S-433). The pyrolysis and GC-MS interfaces were kept at 280°C and the GC-MS was temperature-programmed from 40°C (1 min) to 130°C at 6°C/min, then from 130 to 250°C at 12°C/min and finally from 250 to 300°C at +30°C/min (3 min at 300°C). The MS was operated in the electron impact mode (70 eV) for m/z 40 to 450. The various phenolic pyrolysis compounds were identified by comparison to the spectra of authentic compounds or to published spectra [28].

### Polyacrylamide gel electrophoresis of RG-II

Ten microgrammes of extractive-free samples of mature stems were treated with 1 M Na_2_CO_3_ at 4°C for 16 h, then rinsed with water until neutral pH was achieved. The pellet was suspended in 1 mL ammonium acetate buffer (50 mM, pH 4.8) with 5 U endo-polygalacturonase from *Aspergillus aculeatus* (Megazyme) and incubated at 37°C for 16 h. Solubilized material was dried and re-suspended in 25 μL of ammonium acetate buffer. Polyacrylamide gel electrophoresis and the silver staining were performed according to [29].

### Transcriptomic analysis

Microarray analysis was performed on complete arabidopsis transcriptome containing 24,576 GSTs corresponding to 22,089 genes from arabidopsis [30]. (Hilson et al., 2004). Three independent biological replicates were performed. For each biological replicate we pooled stems from 4 to 6 plants to generate each sample. We collected the first 2 cm at the base of a 20 cm-long floral stems. The plants were grown in a greenhouse under long-day conditions. Total RNA was extracted with the RNeasy Plant Kit (Qiagen) according to the manufacturer’s instructions. For each comparison, one technical replicate with fluorochrome reversal was performed for each biological replicate (i.e., four hybridizations per comparison). We labeled cRNAs with Cy3-dUTP or Cy5-dUTP (Perkin-Elmer-NEN Life Science Products) and performed hybridization and scanning of the slides as previously described [31]. (Lurin et al., 2004).

### Statistical analysis of microarray data

Experiments were designed with the statistics group of the Plant Genomics Research Unit. Statistical analysis was performed with normalization based on dye swapping (i.e., four arrays, each containing 24,576 GSTs and 384 controls) as previously described [32]. For the identification of differentially expressed genes, we performed a paired *t* test on ratios, assuming that the variance of the ratios was similar for all genes. Spots with extreme variances (too small or too large) were excluded. The raw P values were adjusted by the Bonferroni method, which controls the family-wise error rate (with a type I error equal to 5%) to minimize the number of false positives in a multiple-comparison context [33]. We considered genes with a Bonferroni P value ≤ 0.05 to be differentially expressed, as previously described [32].

Microarray data from this article were deposited at Gene Expression Omnibus (http://www.ncbi.nlm.nih.gov/geo/), accession no. GSE 74857) and at CATdb (http://urgv.evry.inra.fr/CATdb/; Project: ELIM1) according to the “Minimum Information About a Microarray Experiment” standards.

### Coexpression network

The edge force directed coexpression networks were generated with Cytoscape 2.8 (http://www.cytoscape.org) from data retrieved from ATTED-II [34].

### Histology

Fresh hand-cut sections were subjected to histochemical analysis (described below) and photographed through with a Zeiss AxioPlan 2 microscope system with automatic exposure. Wiesner staining was performed by incubating sections in 1% phloroglucinol in ethanol:water (7:3) with 30% HCl. Mäule staining was performed by first incubating sections in KMnO_4_ (1%). After 10 min, sections were washed and acidified with HCl (30%) for 1 min, washed again, and then incubated in NaHCO_3_ (5%).

## Results

### *MUR1* mutation alters lignin structure

To explore the role of RG-II dimers on lignin deposition, we performed biochemical analysis on mature *mur1-1* inflorescence. The *mur1-1* mutants are shorter than wild type (S1 Fig) and consequently, accumulate less biomass in inflorescence stems, having with more than a 50% reduction of dry weight compared with wild type. *MUR1* is thereby involved in inflorescence stem development (Table 1) but the lignin content of extractive-free stem material was not affected (Table 1). The neutral sugar composition of the wall was analyzed and, as expected, the amount of fucose residues in *mur1-1* mutants was dramatically reduced [35] (Table 2), but no other significant differences for the other sugars were observed. Subsequent analysis of cellulose content showed no significant differences between *mur1-1* and wild type (Table 2).

**Table 1:** Impact of the *MUR1* mutation on the dry weight and lignin content of mature inflorescence stems.

**Line**	Stem dry weight (mg)	% Klason lignin
*mur1-1*	106.5 ± 13.6*	18.0 (17.6; 18.6)
Wild type	241.7 ± 20.4	18.3 (18.1; 19.0)

The stem dry weight per individual plant was measured. Values are means ± SD from 16 plants separately analyzed. The lignin content is expressed as Klason lignin calculated as weight percentage of the extractive-free sample. Values are medians of three replicates (pools of eight plants), with the minimum and maximum given in parentheses.

* indicates significant differences (one-way ANOVA) compared to the wild-type value at P<0.001

**Table 2:** Monosaccharide composition of alcohol insoluble residue isolated from mature stems.

Sugar	Wild type	*mur1-1*
Sugars from trifluoroacetic hydrolysis (in μg.g^-1^ AIR)		
Fuc	2.2 (2.2; 2.5)	**0.2 (0.2; 0.3)** *
Rha	6.8 (6.7; 7)	8.1 (7.6; 8.3)
Ara	9.4 (8.9; 9.5)	9.7 (9.1; 10.1)
Gal	16.0 (15.6; 16.6)	16.2 (15.3; 17.1)
Glc	5.0 (4.8; 5.2)	5.2 (5.1; 5.8)
Xyl	96.3 (94.9; 100.5)	90.1 (89.5; 95.8)
Man	5.1 (5.1; 5.3)	5.6 (5.2; 5.9)
Sugar from subsequent H_2_SO_4_ hydrolysis		
Glc	324.0 (319.7; 345.9)	304.9 (291.5; 314.0)

Two successive hydrolyses were performed. The first trifluoroacetic hydrolysis released sugars from non cellulosic polysaccharides and the second H_2_SO_4_ hydrolysis released glucose from cellulose. Values are medians of three replicates, with the minimum and maximum given in parentheses.

* indicates a significant difference (Kruskal Wallis test) compared to the wild-type value at P<0.05.

Lignin structure was first evaluated by thioacidolysis. This method provides hydroxy-phenyl, guaiacyl, and syringyl thioethylated monomers from the hydroxy-phenyl, guaiacyl, and syringyl units that are only connected with labile β–*O*–4 bonds in lignin [36]. When expressed relative to the lignin content, the yield of lignin-derived thioacidolysis monomers is reduced when the frequency of resistant inter unit bonds in the lignin polymer is increased. In *mur1-1*, the thioacidolysis yield of mutant stems was significantly reduced by 17% compared with wild type (Table 3). Despite we found a lower frequency of lignin-derived syringyl compounds in *mur1-1* with analytical pyrolysis (S1 Table), the relative frequency of hydroxy-phenyl, guaiacyl, and syringyl thioacidolysis monomers was not substantially affected.

**Table 3:** Determination of thioacidolysis monomers released from extractive-free mature stems.

**Line**	Thioacidolysis monomers (μmol.g^-1^ lignin)	% hydroxy-phenyl	% guaiacyl	% syringyl
*mur1-1*	1004 (942; 1071)*	1.1 (1.1; 1.3)	70.9 (70.4; 72.5)	27.9 (26.4;28.5)
Wild type	1203 (1105; 1322)	1.1 (1.0; 1.2)	70.6 (10.5; 70.9)	28.3 (27.8; 28.4)

Values are medians of three pools of eight plants separately analysed, with the minimum and maximum given in parentheses. Thioacidolysis yields are expressed in μmol.g^-1^ of lignin (measured as Klason lignin).

* indicates a significant difference (Kruskal Wallis test) compared to the wild type value at P<0.05.

### Genes involved in the formation of lignified tissues are overexpressed in *mur1-1*

To identify the genes involved in the structural changes in *mur1-1* lignin, we carried out a transcriptomic analysis with complete Arabidopsis transcriptome microarray (CATMA) chips [30]. This experiment was performed using the basal part of *mur1-1* and wild-type stems at growth stage 6.1, according to [37] and revealed that 1168 genes were differently expressed in *mur1-1* compared to wild type (583 down and 585 up).

**Fig 2:**
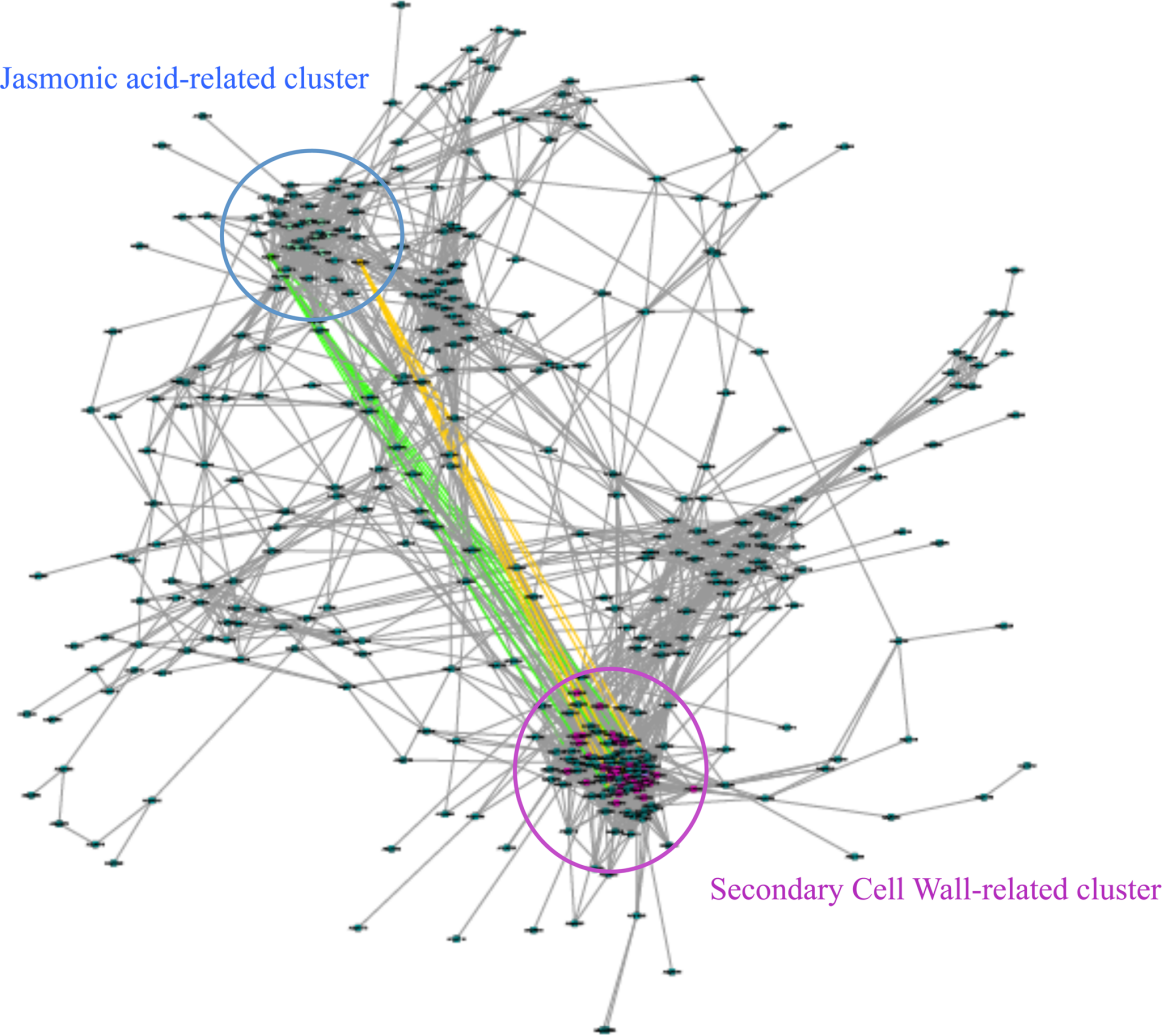
Gene coexpression network made from genes up-regulated in *mur1-1* stems. An edge weighted, force-directed approach was used, based on data retrieved from ATTED-II and visualised in Cytoscape 2.8 (http://www.cytoscape.org). In this network, each gene is represented by a node and each grey edge connecting two nodes represents a mutual rank <100. Two modules of high density were revealed suggesting a tight coexpression. Green and orange edges link *MYB73* and *DDP1* respectively to secondary cell wall genes potentially regulated by these transcription factors (Taylor-Teeples et al., 2014).

Using cytoscape tool (http://www.cytoscape.org), co-expression analysis of down-regulated genes in *mur1-1* did not reveal any obvious co-expression cluster. By contrast, co-expression analysis of up-regulated genes showed four main clusters (Fig 2). Analysis of putative gene functions within the two smallest ones did not reveal any clear correlation with biological processes.

The biggest cluster contained 93 genes, of which 64 are known to be related to secondary cell wall deposition or xylem formation. Among them, one is involved in monolignol synthesis and six in lignin polymerization. We found at least ten genes involved in xylan synthesis and three genes involved in secondary cell wall cellulose synthesis. We also observed at least six transcription factors known to control secondary cell wall synthesis like MYB46 and MYB58 (S2 Table). Notably, the vascular NAC-domain 6 (VND6) master regulatory transcription factor that controls vascular development was also found in this cluster (S2 Table).

The second cluster comprised 42 genes, of which 15 are related to jasmonic acid signaling, a regulator of wound response in vascular plants [38]. Thus, we found in this cluster seven jasmonic acid-responsive genes encoding JASMONATE-ZIM domain proteins, which act as negative regulators of jasmonic acid response [39] (S3 Table), Three genes associated with jasmonic acid production [40],[41],[42] and four members of the ethylene response factor (ERF) family involved in the jasmonic acid-responsive gene expression [39]. One ERF member, RAP2.6 (ERF/ AP2 transcription factor family) is closely related to RAP2.6L which is involved, like the lipooxygenase 2 (LOX2), in tissue repair [43],[44]. Furthermore, the *DDF1* (Dwarf and Delayed Flowering 1) and *MYB73* transcription factors present in this cluster have already been described as candidate regulatory genes for wood formation by interacting with the promoters of several secondary cell wall related genes [45],[46].

### *MUR1* mutation alters inflorescence stem development

**Fig 3:**
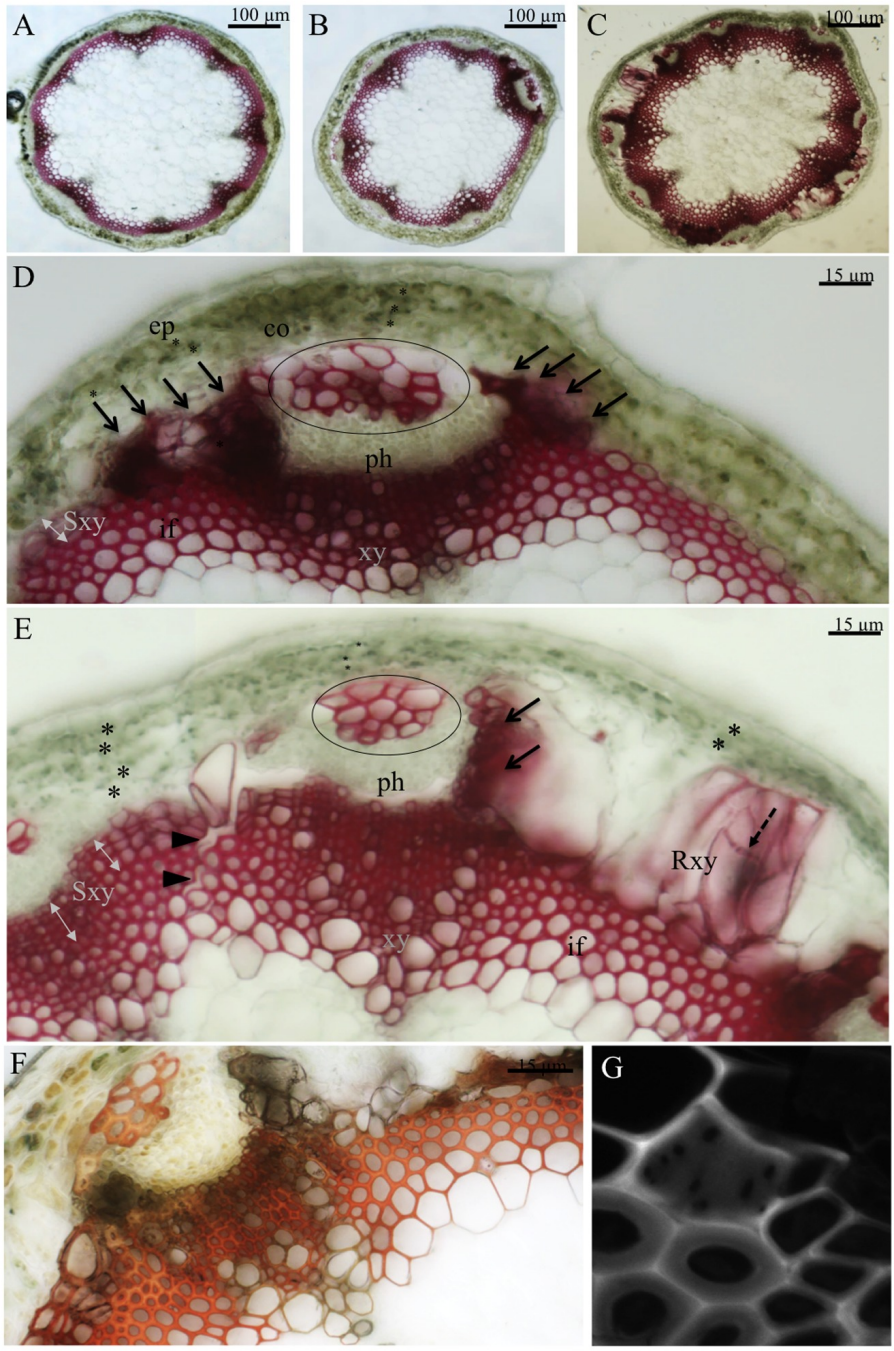
Inflorescence cross sections at intermediate and mature stages of development. Cross sections through the basal region of the infloresence stem for wild type (A) and *mur1-1* (B,D) at an intermediate stage of development. Cross sections of the basal region of the stem of *mur1-1* at a mature stage of development (C,E) stained with phloroglucinol-HCl or with the Maüle reagent (F). Phloem sclereids are encircled on D and E and abnormal lignified cells rich in aldehyde compounds are indicated by black arrows. The pitted cell wall of regenerative xylem and the fragmentation of the sclerenchyma cylinder are indicated by black arrow heads and dotted arrows respectively. The cortex cell wall layer are indicated by asterisks. Observation of the perforation plate of a regenerative xylem cell observed by confocal microscopy (G). Ep: epidermis, co: cortex, ph: phloem, xy: xylem, Sxy: secondary xylem, Rxy: regenerative xylem, if: interfascicular fibers.

The high levels of secondary cell wall related transcripts and particularly the upregulation of VND6 controlling metaxylem vessel formation were intriguing. To build on the biochemical and transcriptomic analysis of *mur1-1* mutants, we performed histological analysis on inflorescence stem cross sections at various development stages. At an intermediate developmental stage (stem of about 15–20 cm, growth stage 6.1 according to [37]), collapsed tracheary elements were observed and fibre-sclereids cells appeared in many phloem poles in *mur1-1* stems whereas they were rarely observed in the wild type (Fig 3). Furthermore, extensive secondary growth and the formation of large lignified and non-lignified cells were observed in the cortical tissues adjacent to vascular bundles (Fig 3). Their occurrence was accompanied with a reduced number of cell layers in the cortex (4–5 cell layers above sclerenchyma against only 3 above ectopic lignified cells).

In mature stems (>25 cm, growth stage 6.9 according to [37]), high fascicular and interfascicular cambial activities were also observed and the phenotypes observed in intermediate developmental stages were more obvious (Fig 3C and 3E). The aberrant cells formed around the phloem tissues exhibited a pitted cell wall (dotted arrow in Fig 3) and they often formed a supplemental layer separating the cortex from the stele. When stained with the Maüle reagent, sections showed brown coloration consistent with a guaiacyl-rich cell wall that is typically observed in tracheary elements (Fig 3). Further examination using confocal microscopy facilitated the observation of perforation plates that are characteristic of tracheary elements (Fig 3).

Finally, many stem cross sections had breaks within the sclerenchymatous cylinder that typically runs through the middle lamellae of neighboring sclerenchymatous cells (Fig 3). The presence of the breaks was often accompanied with the development of a big lignified cell. It is worth noting that similar results were also obtained on *mur1-2*, a second *MUR1* allele descending from an independent mutagenesis event (S2 Fig, [35]).

### The defect of RG-II dimerization might be responsible for phenotypic traits observed in *mur1*

Fucose residues occur not only in RG-II [47] and RG-I [48], but also in xyloglucan [49] and arabinogalactan proteins [50]. However, the tensile strength of *mur1-1* in the elongation zone of the inflorescence stem was completely rescued by the addition of boron [21] thus demonstrating that the lack of fucose in RG-II rather than in xyloglucan, RG-I, or arabinogalactan proteins is important for the weakness of mur1-1 stems. In an attempt to determine the role of reduced boron-dependent RG-II dimerization in the *mur1-1* stem inflorescence, we first confirmed that under our growth conditions, RG-II cross linking is reduced in *mur1-1* mature stems (S3 Fig), second we sprayed *mur1-1* with a boric acid solution as soon as the inflorescence emerged from the rosette, and repeated the spraying twice a week. We then made cross-sections of the mature stems. Despite the boric acid application reducing ectopic lignification and tissue detachment, these phenotypes were not fully rescued (S4 Fig).

**Fig 4:**
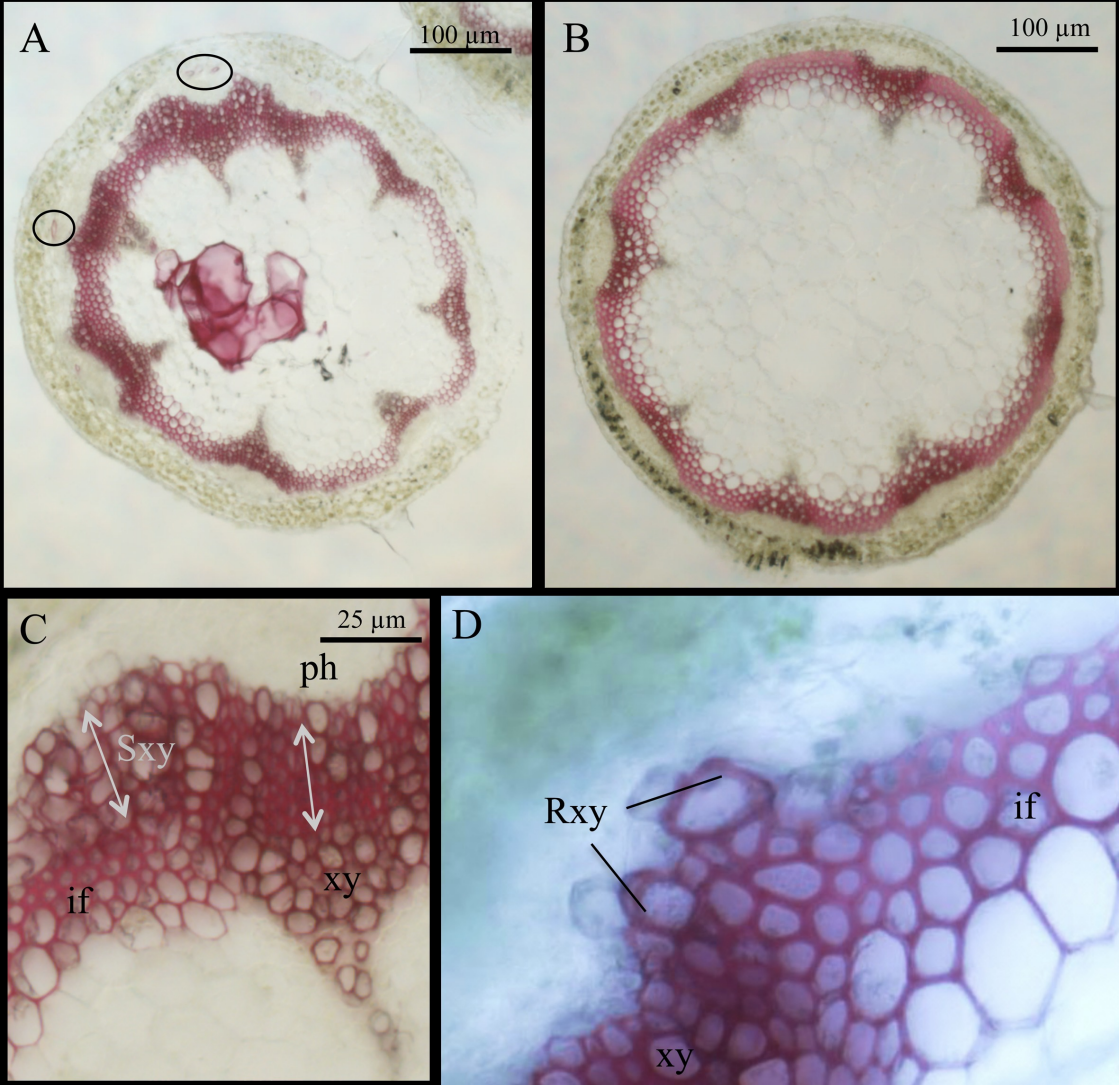
*Bor1-3* mutants have enhanced secondary growth and regenerative xylem. Basal cross-sections of primary inflorescence stained with phloroglucinol-HCl of *bor1-3* (A,C and D) and wild type (B). Phloem sclereids are encircled in A. ph: phloem, xy: xylem, Sxy: secondary xylem, Rxy: regenerative xylem, if: interfascicular fibers.

Because boron penetration might be limited by the cuticule, we decided to study *bor1-3*, a mutant altered in boron xylem loading. However, by contrast to *mur1-1*, this mutant has lost apical dominance [22]. This made it difficult to collect mature inflorescence stems comparable to *mur1-1* and wild type and consequently, we did not run biochemical experiments on this mutant. Nevertheless, we performed cross sections on *bor1-3* and observed that, at an intermediate stage of development, *bor1-3* had, like *mur1-1*, phloem sclereids and abnormal secondary growth (Fig 4). Some regenerative xylem cells were also observed between stele and cortex (Fig 4) and phloroglucinol positive cells were also sometimes present in the pith parenchyma of the *bor1-3* stem (Fig 4). At a later stage of development, the phenotypes did not become evidently more severe, perhaps because of the apical dominance loss (floral stems remained small at mature stage) [22]. Although *bor1-3* is not known to be affected in cell wall composition, we hypothesize that the abnormal secondary growth is caused by reduced dimerization of RG-II owing to limited boron supply [51]. Although we cannot completely exclude a role for the fucose residues in xyloglucan or arabinogalactan proteins, the similarity of secondary cell wall disruptions in *mur1-1* and *bor1-3* taken together with the partial rescue of this phenotype in *mur1-1* by spraying the stem with boric acid lead us to conclude that RGII dimerization plays a pivotal role in the development of tissues containing secondary cell walls.

## Discussion

Analysis of lignin from mature *mur1-1* inflorescence stems revealed that the thioacidolysis yield of mutant stems was significantly reduced compared with wild type. This suggests that lignin in *mur1-1* is enriched in resistant inter-unit linkages, which are more abundant in lignin formed in response to stress [52]. Nevertheless, polymerization of lignin is affected by the polysaccharidic matrix, at least based on *in vitro* experiments [53]. Therefore, we hypothesize that lignin polymerization in *mur1-1* is altered by changed pectin composition or reduced RG-II cross linking. Supporting this hypothesis, RG-II is enriched at cell corners (Fig 4) where nucleation sites of lignification are also found [54],[4].

Microarrays analysis performed on bottom part of *mur1-1* inflorescence showed that disrupting GDP-fucose synthesis is correlated with lignification and hormone signaling. For instance, *mur1-1* co-overexpresses jasmonate-responsive genes with two transcription factors, *DDF1* (Dwarf and Delayed Flowering 1) and *MYB73* previously identified as candidate regulatory genes for wood formation [45],[46]. These data support the idea that first, *mur1-1* plants synthesized more secondary cell wall than wild type in the basal part of the stem and, second, that the jasmonic acid signaling pathway participates in the overexpression of secondary cell wall-related genes through the DDF1 and MYB73 transcription factors. Furthermore, this is consistent with the fact that jasmonic acid was found to trigger ectopic lignification [55]. Indeed, histological analysis revealed the unusual formation of large cells in the cortical tissues adjacent to vascular bundles in *mur1-1* and *mur1-2* mutants. When subjected to lignin staining reagents the wall of these cortex cells showed typical lignin color found in vessel walls, which are known to be poor in S-unit of lignin. Together, these findings support the idea that these cells are regenerative xylem cells [56].

**Fig 5:**
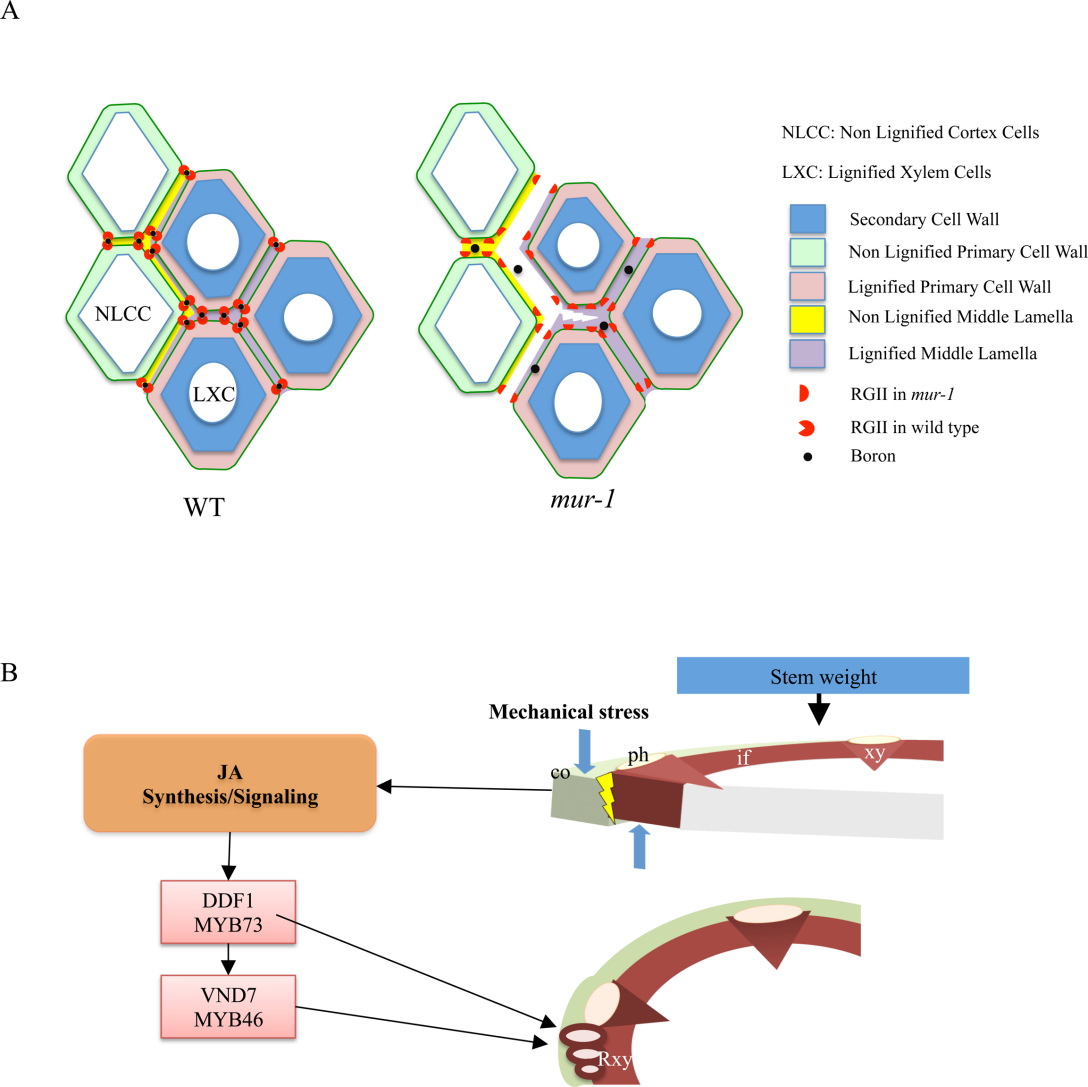
Putative mechanisms responsible for the *mur1-1* phenotype in the inflorescence stem. **A.** Diagram of cell adhesion deficiency at the edge of lignified tissues and non-lignified cortex cells in *mur1-1*. RG-II dimers are enriched in the cell corners. Relative sizes of middle lamella, primary and secondary cell walls are not drawn to scale. **B.** Working hypothesis of regenerative xylem formation in the *mur1-1* inflorescence stem. The cell adhesion is compromised by reduced RG-II cross linking. Mechanical stress on cortex and lignified tissues induces loss of cell adhesion and then transcription of jasmonate related genes. Regenerative elements are formed by activation of specific transcription factors. Co: cortex, ph: phloem, xy: xylem, Rxy: regenerative xylem, if: interfascicular fibers. Blue arrows: shear forces.

The ectopic formation of tracheary elements presumably necessitates induction of master transcriptional regulators genes to drive cell differentiation. The overexpression of the vascular NAC-domain 6 (*VND*) transcription factor in *mur1-1* compared with wild type is in accordance with this phenomenon. Up to now, the formation of regenerative xylem has always been described after the occurrence of wounding [56]. Insofar as wounding induces the jasmonate pathway, this is consistent with the overexpression of jasmonic acid-related genes in *mur1-1*. Mechanical stresses that increase with stem weight could induce a break of the innermost cortical cells from the sclerenchyma in *mur1-1* and *mur1-2* plants (Fig 5). This detachment could be interpreted by the plant as a wound and would therefore trigger the development of regenerative xylem, likely through a jasmonic acid signaling pathway (Fig 5). It is worth noting that the ectopically formed xylem-like cells in the cortex are mainly detectable at the interface of cortex, vascular bundles, and interfascicular sclerenchyma. These three tissues have distinct structures and mechanical properties due to different cell shape and cell wall composition. This may indicate a reduced resistance to shear forces induced by bending stress in fucose deficient plants and that the cell adhesion involving lignified cells is altered in *mur1-1* and *mur1-2* plants (Fig 5). The presence of regenerative xylem in *mur1-1* and *mur1-2* stems is consistent with the enrichment of resistant linkages in lignin, a structural trait already described for stress lignin [52]. Finally, the ectopic lignification is specific to *mur1-1* because other mutants altered in homogalacturonan biosynthesis with a severe defect in cell adhesion such as *quasimodo2*, do not display such a phenotype [57] (Fuentes et al., 2010, and S2 Fig).

## Supporting information

S1 Fig Photographs of mature *mur1-1* and wild-type plants. (PDF)

S2 Fig Hand-cut sections of mature stems from *mur1-1*, *mur1-2*, *qua2*, and wild type (WT) stained with phloroglucinol-HCl. (PDF)

S3 Fig Polyacrylamide gel electrophoresis of RG-II. (PDF)

S4 Fig Cross-sections of mature stems from wild type (WT) and *mur1-1* stained with phloroglucinol-HCl. The *mur1-1* mutant was sprayed with a 5mg/mL boron (B) solution.
(PDF)

S1 Table Relative frequencies of lignin-derived Guaiacyl and Syringyl pyrolysis products from extractive-free mature stems. * indicates significant differences (Kruskal Wallis test) compared to the wild-type value at P<0.05.
(PDF)

S2 Table Secondary cell wall related genes over-expressed in *mur1-1*. (PDF)

S3 Table List of jasmonic acid-related genes overexpressed in *mur1-1*. (PDF)

## References

[1] Daher FB, Braybrook SA. How to let go: pectin and plant cell adhesion. Front Plant Sci. 2015 doi: 10.3389/fpls.2015.00523 26236321 10.3389/fpls.2015.00523 PMC4500915

[2] Adler E. Lignin chemistry-past, present and future. Wood Sci Technol. 1977;11: 169–218.

[3] Boerjan W, Ralph J, Baucher M. Lignin biosynthesis. Annu Rev Plant Biol. 2003;54: 519–546. doi: 10.1146/annurev.arplant.54.031902.134938 14503002 10.1146/annurev.arplant.54.031902.134938

[4] Terashima N, Fukushima K, He L-F, Takabe K Comprehensive model of the lignified plant cell wall. In: Forage Cell Wall Structure and Digestibility (HG Yung et al, ed) ASACSSA-SSSA. 1993. pp. 247–270.

[5] Donaldson LA. Lignification and lignin topochemistry—an ultrastructural view. Phytochemistry. 2001;57: 859–873. 11423137 10.1016/s0031-9422(01)00049-8

[6] Jarvis MC. Structure and properties of pectin gels in plant cell walls. Plant Cell Environ. 1984;7: 153–164.

[7] O’Neill MA, Ishii T, Albersheim P, Darvill AG. Rhamnogalacturonan II: structure and function of a borate cross-linked cell wall pectic polysaccharide. Annu Rev Plant Biol. 2004;55: 109–139. doi: 10.1146/annurev.arplant.55.031903.141750 15377216 10.1146/annurev.arplant.55.031903.141750

[8] Ndeh D, Rogowski A, Cartmell A., Luis AS, Basle A, Gray J et al Complex pectin metabolism by gut bacteria reveals novel catalytic functions. Nature. 2017;544, 65–70. doi: 10.1038/nature21725 28329766 10.1038/nature21725 PMC5388186

[9] O’Neill MA, Warrenfeltz D, Kates K, Pellerin P, Doco T, Darvill AG et al Rhamnogalacturonan-II, a pectic polysaccharide in the walls of growing plant cell, forms a dimer that is covalently cross-linked by a borate ester. In vitro conditions for the formation and hydrolysis of the dimer. J Biol Chem. 1996;271: 22923–22930. 8798473 10.1074/jbc.271.37.22923

[10] Lewis DH. Boron, lignification and the origin of vascular plants—A unified hypothesis. New Phytol. 1980;84: 209–229.

[11] Matsunaga T. Occurrence of the primary cell wall polysaccharide rhamnogalacturonan II in Pteridophytes, Lycophytes, and Bryophytes. Implications for the evolution of vascular plants. Plant Physiol. 2004;134: 339–351. doi: 10.1104/pp.103.030072 14671014 10.1104/pp.103.030072 PMC316313

[12] Popper Z. Evolution and diversity of green plant cell walls. Curr Opin Plant Biol. 2008;11: 286–292. doi: 10.1016/j.pbi.2008.02.012 18406657 10.1016/j.pbi.2008.02.012

[13] Pabst M, Fischl RM, Brecker L, Morelle W, Fauland A, Köfeler H et al Rhamnogalacturonan II structure shows variation in the side chains monosaccharide composition and methylation status within and across different plant species. Plant J Cell Mol Biol. 2013;76: 61–72. 10.1111/tpj.12271 23802881

[14] O’Neill MA, Eberhard S, Albersheim P, Darvill AG. Requirement of borate cross-linking of cell wall rhamnogalacturonan II for Arabidopsis growth. Science. 2001;294: 846–849. doi: 10.1126/science.1062319 11679668 10.1126/science.1062319

[15] Ahn JW, Verma R, Kim M, Lee JY, Kim YK, Bang JW et al Depletion of UDP-D-apiose/UDP-D-xylose synthases results in rhamnogalacturonan-II deficiency, cell wall thickening, and cell death in higher plants. J Biol Chem. 2006;281: 13708–13716. doi: 10.1074/jbc.M512403200 16549428 10.1074/jbc.M512403200

[16] Delmas F, Séveno M, Northey JGB, Hernould M, Lerouge P, McCourt P et al (2008) The synthesis of the rhamnogalacturonan II component 3-deoxy-D-*manno*-2-octulosonic acid (Kdo) is required for pollen tube growth and elongation. J Exp Bot. 2008;59: 2639–2647. doi: 10.1093/jxb/ern118 18503041 10.1093/jxb/ern118 PMC2486460

[17] Voxeur A, Gilbert L, Rihouey C, Driouich A, Rothan C, Baldet P et al Silencing of the GDP-D-mannose 3,5-epimerase affects the structure and cross-linking of the pectic polysaccharide rhamnogalacturonan II and plant growth in tomato. J Biol Chem. 2011;286: 8014–8020. doi: 10.1074/jbc.M110.198614 21224383 10.1074/jbc.M110.198614 PMC3048688

[18] Dumont M, Lehner A, Bouton S, Kiefer-Meyer MC, Voxeur A, Pelloux J et al The cell wall pectic polymer rhamnogalacturonan-II is required for proper pollen tube elongation: implications of a putative sialyltransferase-like protein. Ann Bot. 2014;114: 1177–1188. doi: 10.1093/aob/mcu093 24825296 10.1093/aob/mcu093 PMC4195553

[19] Reiter WD, Chapple C, Somerville CR. Altered growth and cell walls in a fucose-deficient mutant of Arabidopsis. Science. 1993;261: 1032–1035. doi: 10.1126/science.261.5124.1032 17739625 10.1126/science.261.5124.1032

[20] Reuhs BL, Glenn J, Stephens SB, Kim JS, Christie DB, Glushka JG et al L-Galactose replaces L-fucose in the pectic polysaccharide rhamnogalacturonan II synthesized by the L-fucose-deficient *mur1* Arabidopsis mutant. Planta. 2004;219: 147–157. doi: 10.1007/s00425-004-1205-x 14991405 10.1007/s00425-004-1205-x

[21] Ryden P, Sugimoto-Shirasu K, Smith AC, Findlay K, Reiter WD, McCann MC. Tensile properties of Arabidopsis cell walls depend on both a xyloglucan cross-linked microfibrillar network and rhamnogalacturonan II-borate complexes. Plant Physiol. 2003;132, 1033–1040. doi: 10.1104/pp.103.021873 12805631 10.1104/pp.103.021873 PMC167041

[22] Takano J, Noguchi K, Yasumori M, Kobayashi M, Gajdos Z, Miwa K et al Arabidopsis boron transporter for xylem loading. Nature. 2002;420: 337–340. doi: 10.1038/nature01139 12447444 10.1038/nature01139

[23] Miwa K, Wakuta S, Takada S, Ide K, Takano J, Naito S et al Roles of BOR2, a boron exporter, in cross linking of rhamnogalacturonan II and root elongation under boron limitation in Arabidopsis. Plant Physiol. 2013;163: 1699–1709. doi: 10.1104/pp.113.225995 24114060 10.1104/pp.113.225995 PMC3850200

[24] Harholt J, Jensen JK, Sørensen SO, Orfila C, Pauly M, Scheller HV. ARABINAN DEFICIENT 1 is a putative arabinosyltransferase involved in biosynthesis of pectic arabinan in Arabidopsis. Plant Physiol. 2006;140: 49–58. doi: 10.1104/pp.105.072744 16377743 10.1104/pp.105.072744 PMC1326030

[25] Updegraff DM. Semimicro determination of cellulose in biological materials. Anal Biochem. 1969;32: 420–424. 5361396 10.1016/s0003-2697(69)80009-6

[26] Dence CW. The determination of lignin *In* Lin SY, Dence CW, eds, *Methods in Lignin Chemistry*. Springer Berlin Heidelberg, Berlin, Heidelberg, 1992 pp. 33–61.

[27] Lapierre C, Pollet B, Petit-Conil M, Toval G, Romero J, Pilate G et al Structural alterations of lignins in transgenic poplars with depressed cinnamyl alcohol dehydrogenase or caffeic acid *O*-methyltransferase activity aave an opposite impact on the efficiency of industrial kraft pulping. Plant Physiol. 1999;119: 153–164. 9880356 10.1104/pp.119.1.153 PMC32214

[28] Ralph J and Hatfield D. Pyrolysis-GC-MS characterization of forage materials. J Agric Food Chem. 1991;39: 1428–1437.

[29] Chormova D, Messenger DJ, Fry SC (2014) Boron bridging of rhamnogalacturonan-II,monitored by gel electrophoresis, occurs during polysaccharide synthesis and secretion but not post-secretion. Plant J 77: 534–46 doi: 10.1111/tpj.12403 24320597 10.1111/tpj.12403 PMC4171739

[30] Hilson P, Allemeersch J, Altmann T, Aubourg S, Avon A, Beynon J et al (2004) Versatile gene-specific sequence tags for Arabidopsis functional genomics: transcript profiling and reverse genetics applications. Genome Res 14: 2176–2189 doi: 10.1101/gr.2544504 15489341 10.1101/gr.2544504 PMC528935

[31] Lurin C, Andrés C, Aubourg S, Bellaoui M, Bitton F, Bruyère C et al (2004) Genome-wide analysis of Arabidopsis pentatricopeptide repeat proteins reveals their essential role in organelle biogenesis. Plant Cell 16: 2089–2103 doi: 10.1105/tpc.104.022236 15269332 10.1105/tpc.104.022236 PMC519200

[32] Gagnot S, Tamby JP, Martin-Magniette ML, Bitton F, Taconnat L, Balzergue S et al (2008) CATdb: a public access to Arabidopsis transcriptome data from the URGV-CATMA platform. Nucleic Acids Res 36: 986–990 10.1093/nar/gkm757 PMC2238931 17940091

[33] Ge Y, Dudoit S, Speed TP (2003) Resampling-based multiple testing for microarray data analysis. Test 12: 1–77

[34] Obayashi T, Kinoshita K (2010) Coexpression landscape in ATTED-II: usage of gene list and gene network for various types of pathways. J Plant Res 123: 311–319 doi: 10.1007/s10265-010-0333-6 20383554 10.1007/s10265-010-0333-6

[35] Reiter WD, Chapple C, Somerville CR (1997) Mutants of *Arabidopsis thaliana* with altered cell wall polysaccharide composition. Plant J Cell Mol Biol 12: 335–345 10.1046/j.1365-313x.1997.12020335.x 9301086

[36] Rolando C, Monties B, Lapierre C (1992) Thioacidolysis *In* Lin SY, Dence CW, eds, *Methods in Lignin Chemistry*. Springer Berlin Heidelberg, Berlin, Heidelberg, pp 334–349

[37] Boyes DC, Zayed AM, Ascenzi R, McCaskill AJ, Hoffman NE, Davis KR et al (2001) Growth stage-based phenotypic analysis of Arabidopsis: a model for high throughput functional genomics in plants. Plant Cell 13: 1499–1510 doi: 10.1105/TPC.010011 11449047 10.1105/TPC.010011 PMC139543

[38] Léon J, Rojo E, Sanchez-Serrano JJ (2001) Wound signalling in plants. J Exp Bot 52: 1–9 10.1093/jexbot/52.354.1 11181708

[39] Santino A, Taurino M, De Domenico S, Bonsegna S, Poltronieri P, Pastor V et al (2013) Jasmonate signaling in plant development and defense response to multiple (a)biotic stresses. Plant Cell Rep 32: 1085–1098 doi: 10.1007/s00299-013-1441-2 23584548 10.1007/s00299-013-1441-2

[40] Bell E, Mullet JE. Characterization of an Arabidopsis lipoxygenase gene responsive to methyl jasmonate and wounding. Plant Physiol. 1993;103: 1133–1137. 8290626 10.1104/pp.103.4.1133 PMC159098

[41] Schaller F, Biesgen C, Müssig C, Altmann T, Weiler EW. 12-Oxophytodienoate reductase 3 (OPR3) is the isoenzyme involved in jasmonate biosynthesis. Planta. 2000;210: 979–984. doi: 10.1007/s004250050706 10872231 10.1007/s004250050706

[42] Caldelari D, Wang G, Farmer EE, Dong X. Arabidopsis *lox3 lox4* double mutants are male sterile and defective in global proliferative arrest. Plant Mol Biol. 2011;75: 25–33. doi: 10.1007/s11103-010-9701-9 21052784 10.1007/s11103-010-9701-9

[43] Reid JB and Ross JJ. Regulation of tissue repair in plants. Proc Natl Acad Sci U S A. 2011;108: 17241–17242. doi: 10.1073/pnas.1114432108 21960442 10.1073/pnas.1114432108 PMC3198324

[44] Asahina M, Azuma K, Pitaksaringkarn W, Yamazaki T, Mitsuda N, Ohme-Takagi et al Spatially selective hormonal control of RAP2.6L and ANAC071 transcription factors involved in tissue reunion in Arabidopsis. Proc Natl Acad Sci U S A. 2011;108: 16128–16132. doi: 10.1073/pnas.1110443108 21911380 10.1073/pnas.1110443108 PMC3179063

[45] Ko J, Han K. Arabidopsiswhole-transcriptome profiling defines the features of coordinated regulations that occur during secondary growth. Plant Mol Biol. 2004;55: 433–453. doi: 10.1007/s11103-004-1051-z 15604691 10.1007/s11103-004-1051-z

[46] Taylor-Teeples M, Lin L, de Lucas M, Turco G, Toal TW, Gaudinier A et al An Arabidopsis gene regulatory network for secondary cell wall synthesis. Nature. 2014;517: 571–575. doi: 10.1038/nature14099 25533953 10.1038/nature14099 PMC4333722

[47] Glushka JN, Terrell M, York WS, O’Neill MA, Gucwa A, Darvill AG et al Primary structure of the 2-*O*-methyl-alpha-L-fucose-containing side chain of the pectic polysaccharide, rhamnogalacturonan II. Carbohydr Res. 2003;338: 341–352. 12559732 10.1016/s0008-6215(02)00461-5

[48] Nakamura A, Furuta H, Maeda H, Nagamatsu Y, Yoshimoto A Analysis of structural components and molecular construction of soybean soluble polysaccharides by stepwise enzymatic degradation. Biosci Biotechnol Biochem. 2001;65: 2249–2258. doi: 10.1271/bbb.65.2249 11758917 10.1271/bbb.65.2249

[49] Lerouxel O, Choo TS, Séveno M, Usadel B, Faye L, Lerouge P et al Rapid structural phenotyping of plant cell wall mutants by enzymatic oligosaccharide fingerprinting. Plant Physiol. 2002;130: 1754–1763. doi: 10.1104/pp.011965 12481058 10.1104/pp.011965 PMC1540271

[50] Wu Y, Williams M, Bernard S, Driouich A, Showalter AM, Faik A. Functional identification of two nonredundant Arabidopsis alpha (1,2) fucosyltransferases specific to arabinogalactan proteins. J Biol Chem. 2010;285: 13638–13645. doi: 10.1074/jbc.M110.102715 20194500 10.1074/jbc.M110.102715 PMC2859526

[51] Noguchi K, Yasumori M, Imai T, Naito S, Matsunaga T, Oda H et al *bor1-1*, an Arabidopsis thaliana mutant that requires a high level of boron. Plant Physiol. 1997; 115: 901–906. 9390427 10.1104/pp.115.3.901 PMC158553

[52] Cabané M, Pireaux JC, Léger E, Weber E, Dizengremel P, Pollet B et al Condensed lignins are synthesized in poplar leaves exposed to ozone. Plant Physiol. 2004;134: 586–594. doi: 10.1104/pp.103.031765 14730080 10.1104/pp.103.031765 PMC344535

[53] Touzel JP, Chabbert B, Monties B, Debeire P, Cathala B. Synthesis and characterization of dehydrogenation polymers in Gluconacetobacter xylinus cellulose and cellulose/pectin composite. J Agric Food Chem. 2003:51, 981–986. doi: 10.1021/jf020200p 12568559 10.1021/jf020200p

[54] Matoh T, Takasaki M, Takabe K, Kobayashi M. Immunocytochemistry of rhamnogalacturonan II in cell walls of higher plants. Plant Cell Physiol.1998;39: 483–491.

[55] Mélida H, Largo-Gosens A, Novo-Uzal E, Santiago R, Pomar F, García P et al Ectopic lignification in primary cellulose-deficient cell walls of maize cell suspension cultures: Lignin in primary cellulose-deficient cell walls. J Integr Plant Biol. 2015;57: 357–372. doi: 10.1111/jipb.12346 25735403 10.1111/jipb.12346

[56] Flaishman MA, Loginovsky K, Lev-Yadun S. Regenerative xylem in inflorescence stems of Arabidopsis thaliana. J Plant Growth Regul. 2003;22: 253–258.

[57] Fuentes S, Pires N, Østergaard L. A clade in the QUASIMODO2 family evolved with vascular plants and supports a role for cell wall composition in adaptation to environmental changes. Plant Mol Biol. 2010;73: 605–615. doi: 10.1007/s11103-010-9640-5 20464626 10.1007/s11103-010-9640-5

