# Supplementary figures and images for: Altered lignification in mur1-1 a mutant deficient in GDP-L-fucose synthesis with reduced RG-II cross linking
Source: PLoS One. 2017 Sep 29;12(9):e0184820. doi: 10.1371/journal.pone.0184820 (PMC5621668; doi:10.1371/journal.pone.0184820)

Figure S1

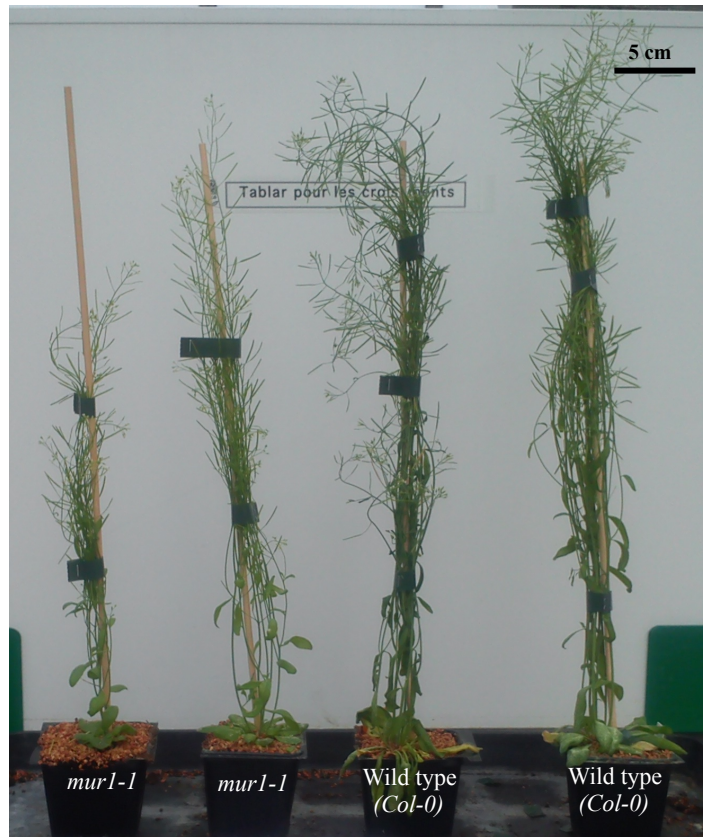

Figure S1 : Phenotype of mature *mur1-1* and wild-type plants.

Supplement: S1 Fig — (PDF) [file pone.0184820.s001.pdf]

Figure S3

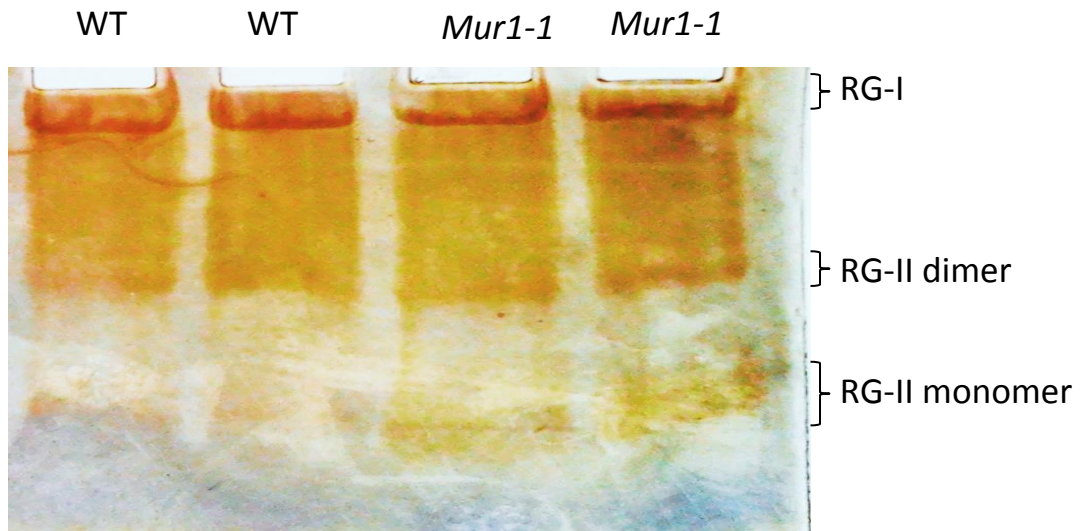

Figure S3 : Polyacrylamide gel electrophoresis of RG-II.

Supplement: S3 Fig — (PDF) [file pone.0184820.s003.pdf]
