# Supplementary material for: Altered lignification in mur1-1 a mutant deficient in GDP-L-fucose synthesis with reduced RG-II cross linking
Source: PLoS One. 2017 Sep 29;12(9):e0184820. doi: 10.1371/journal.pone.0184820 (PMC5621668; doi:10.1371/journal.pone.0184820)
Supplement: S2 Fig — (PDF) [file pone.0184820.s002.pdf]

Figure S2

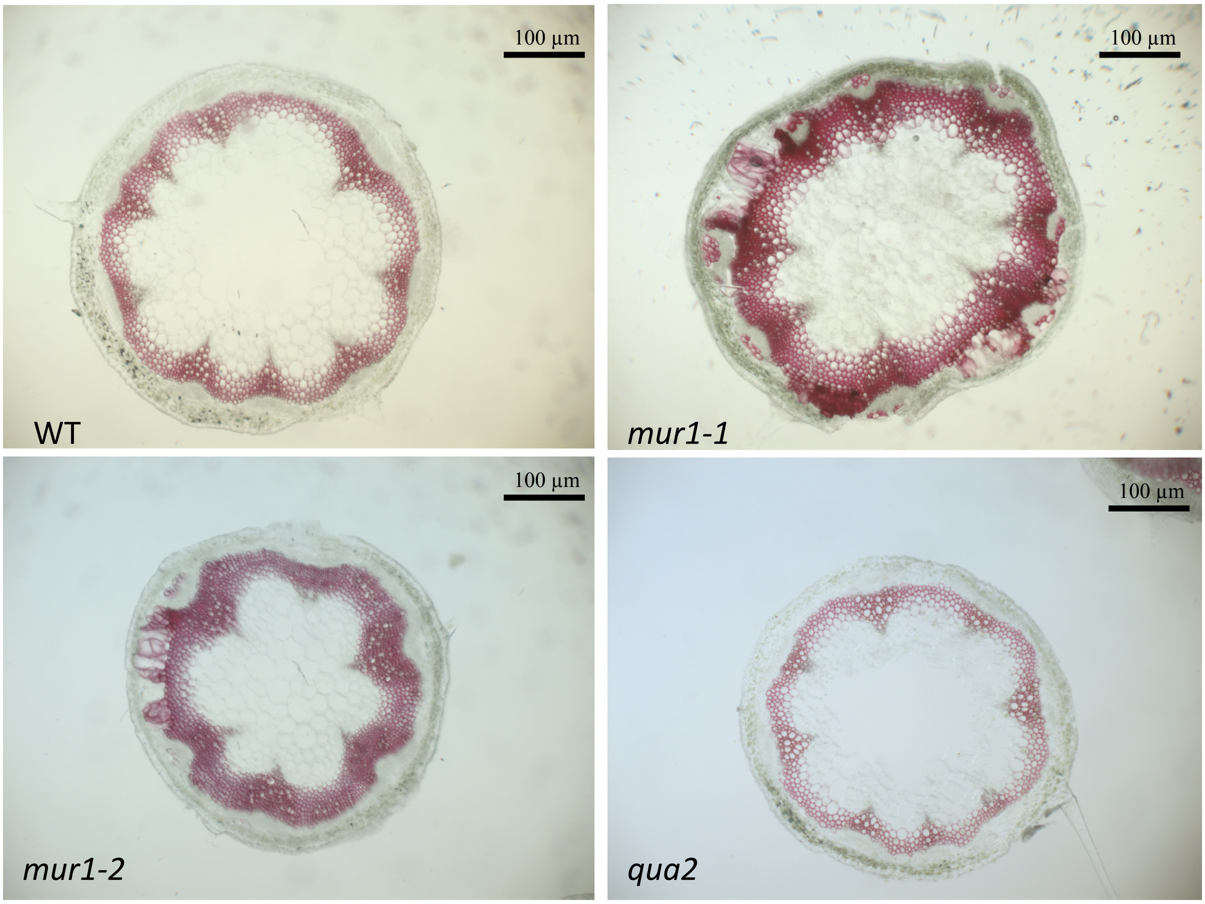

Figure S2 : Hand-cut sections of mature stems from *mur1-1*, *mur1-2*, *qua2* and wild type (WT) stained with phloroglucinol-HCl.
