# Supplementary material for: Altered lignification in mur1-1 a mutant deficient in GDP-L-fucose synthesis with reduced RG-II cross linking
Source: PLoS One. 2017 Sep 29;12(9):e0184820. doi: 10.1371/journal.pone.0184820 (PMC5621668; doi:10.1371/journal.pone.0184820)
Supplement: S4 Fig — The mur1-1 mutant was sprayed with a 5mg/mL boron (B) solution. (PDF) [file pone.0184820.s004.pdf]

Figure S4

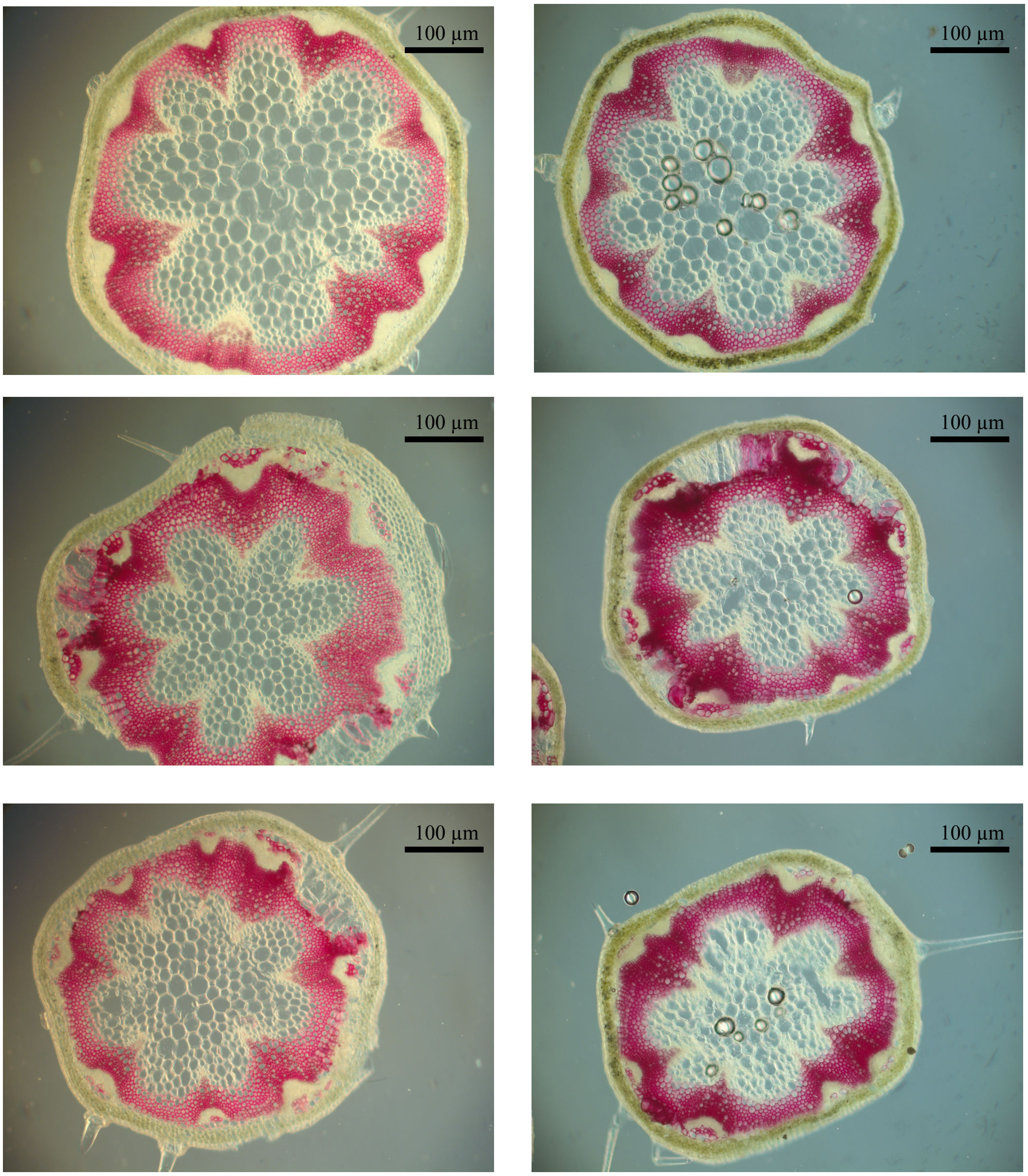

Figure S4 : Cross-sections of mature stems from wild type (WT) and *mur1-1* stained with phloroglucinol-HCl. *mur1-1* was sprayed with a 5mg/mL boric acid (B) solution to rescue the observed phenotype
