# Supplementary material for: Altered lignification in mur1-1 a mutant deficient in GDP-L-fucose synthesis with reduced RG-II cross linking
Source: PLoS One. 2017 Sep 29;12(9):e0184820. doi: 10.1371/journal.pone.0184820 (PMC5621668; doi:10.1371/journal.pone.0184820)
Supplement: S1 Table — * indicates significant differences (Kruskal Wallis test) compared to the wild-type value at P<0.05. (PDF) [file pone.0184820.s005.pdf]

**S1 Table**

**Relative frequencies of lignin-derived Guaiacyl and Syringyl pyrolysis products from extractive-free mature stems.**

| **Line** | **% Guaiacyl** | **% Syringyl** |
|---|---|---|
| ***mur1-1*** | **80.0 (79.9; 81.1)*** | **20.0 (18.9; 20.1)*** |
| **Wild type** | 77.8 (76.0; 78.7) | 22.2 (21.3; 24) |

* indicates significant differences (Kruskal Wallis test) compared to the wild-type value at $P<0.05$.
