# Supplementary material for: Altered lignification in mur1-1 a mutant deficient in GDP-L-fucose synthesis with reduced RG-II cross linking
Source: PLoS One. 2017 Sep 29;12(9):e0184820. doi: 10.1371/journal.pone.0184820 (PMC5621668; doi:10.1371/journal.pone.0184820)
Supplement: S2 Table — (PDF) [file pone.0184820.s006.pdf]

S2 Table

Secondary cell wall related genes over-expressed in *mur1-1*.

**Secondary cell wall**			**Mutant/wild-type (fold change)**
**Lignin**			
At2g29130	LAC2	Putative laccase	2.23
At5g03260	LAC11	Lignin laccase	1.72
At5g05390	LAC12	Putative laccase	3.23
At5g60020	LAC17	Lignin laccase	2.39
At5g42180	PER64	Lignin peroxidase	4.5
At5g51890	PEROXIDASE	Arabidopsis ortholog of ZPO-C	1.78
**Xylan**			
At1g33800	GXM1	Glucuronoxylan methyltranferase	1.96
At4g09990	GXM2	Glucuronoxylan methyltranferase	2.62
At1g09610	GXM3	Glucuronoxylan methyltranferase	2.46
At3g18660	GUX1	Glucuronic acid substitution of xylan	1.82
At1g19300	PARVUS	Glycosyltransferase putative (GT8)	2.00
At2g37090	IRX9	Glycosyltransferase putative (GT43)	2.27
At1g27440	IRX10/GUT2	Glucuronoxylan glucuronosyltransferase	1.71
At5g59290	UXS3	UDP-Glucuronic acid decarboxylase	2.64
At2g28760	UXS6	UDP-Glucuronic acid decarboxylase	1.78
At3g50220	IRX15	DUF579	1.78
At5g17420	MUR10/IRX3/CESA7	Cellulose synthase	2.91

**Cellulose**			
At5g44030	IRX5/CESA4	Cellulose synthase	2.38
At4g18780	CESA8/IRX1	Cellulose synthase	2.35
**Transcription factors**			
At1g32770	SND1	NAC-domain transcription factor	1.97
At4g28500	SND2	NAC-domain transcription factor	1.91
At5g62380	VND6	NAC-domain transcription factor	2.38
At5g12870	MYB46	Member of the R2R3 factor gene family	2.69
At1g16490	MYB58	Member of the R2R3 factor gene family	1.92
At1g63910	MYB103	Positive regulation of syringal lignin biosynthetic process	1.93
**Other**			
At5g54690	IRX8	Putative galacturonosyltransferase	1.96
At5g15630	IRX6/COBL4	Member of the COBRA family	2.01
At4g17220	MAP70-5	Microtubule Associated Protein 70-5	1.88
At1g27920	MAP65-8	Microtubule Associated Protein 65-8	1.75
At5g01360	TBL3	Methyltransferase	2.25
**Xylem formation**			
At4g35350	XCP1	Xylem cysteine peptidase	1.71
At1g20850	XCP2	Xylem cysteine peptidase	2.04
At1g43790	TED6	Tracheary element differentiation-related	1.77
