# Supplementary material for: Altered lignification in mur1-1 a mutant deficient in GDP-L-fucose synthesis with reduced RG-II cross linking
Source: PLoS One. 2017 Sep 29;12(9):e0184820. doi: 10.1371/journal.pone.0184820 (PMC5621668; doi:10.1371/journal.pone.0184820)
Supplement: S3 Table — (PDF) [file pone.0184820.s007.pdf]

S3 Table

List of jasmonic acid-related genes overexpressed in *mur1-1*.

**Jasmonic acid-related**			**Mutant/wild-type (fold change)**
**JASMONATE-ZIM-DOMAIN PROTEIN**			
At1g19180	JAZ1		2.07
At1g74950	JAZ2		2.13
At1g17380	JAZ5		2.58
At2g34600	JAZ7		7.78
At1g30135	JAZ8		5.82
At1g70700	JAZ9		2.57
At5g13220	JAZ10		10.13
**JA biosynthesis**			
At3g45140	LOX2	Lipoxygenase	5.58
At1g72520	LOX4		1.78
At2g06050	OPR3	Oxophytodienoate-reductase	1.79
**Transcription factors**			
At1g43160	RAP2.6	Related to AP2.6	3.2
At1g74930	ORA47	Member of the DREB subfamily A-5 of ERF/AP2 transcription factor family	4.44
At4g17500	ERF1	Ethylene Responsive element binding Factor 1	2.48
At2g44840	ERF13	Ethylene Responsive element binding Factor 13	5.21
At4g34410	RRTF1	Redox Responsive Transcription Factor 1	4.63
At1g12610	DDF1	Dwarf and Delayed Flowering 1	2.36

At1g80840	WRKY40	WRKY DNA-Binding protein 40	2.14
At2g46510	AIB	ABA-Inducible BHLH-type transcription factor	1.8
At3g23250	MYB15	MYB domain protein 15	2.3
At4g37260	MYB73	MYB domain protein 73	1.83
